# Influence of Carbon Content on the Microstructure, Mechanical Properties, Tribological Behavior, and Thermal Stability of (TiAlTaZrNb)Cx High-Entropy Carbide Coatings

**DOI:** 10.3390/ma19153243

**Published:** 2026-07-31

**Authors:** Gilberto Bejarano Gaitán, Daniela María Chimá, Juan Manuel Meza, Aleksei Obrosov, Sabine Weiß

**Affiliations:** 1 Centro de Investigación, Innovación y Desarrollo de Materiales CIDEMAT, Facultad de Ingeniería, Universidad de Antioquia, Calle 67 No. 53-108, Medellín 050010, Colombia; daniela.chima@udea.edu.co; 2 Design of Advanced Composites, Departamento de Materiales y Minerales, Facultad de Minas, Universidad Nacional de Colombia Sede Medellín, Calle 75# 79A-51, Bloque M17, Oficina 407, Medellín 050034, Colombia; jmmezam@unal.edu.co; 3 Department of Physical Metallurgy and Materials Technology, Brandenburg University of Technology Cottbus-Senftenberg, 03046 Cottbus, Germany; aleksei.obrosov@b-tu.de (A.O.); sabine.weiss@b-tu.de (S.W.)

**Keywords:** high-entropy carbide coatings, magnetron sputtering, (TiAlTaZrNb)Cx, mechanical properties, tribology, thermal stability

## Abstract

High-entropy carbide (HEC) coatings have emerged as promising candidates for extreme tribological and high-temperature applications; therefore, the objective of this work is to systematically investigate the correlation between carbon stoichiometry and the microstructural evolution, mechanical response, and thermal stability of (TiAlTaZrNb)Cx high-entropy carbide coatings. Here, HEC coatings were synthesized via reactive unbalanced-field pulsed-bias magnetron sputtering, with methane flow rates precisely tuned to yield carbon concentrations ranging from 24 to 55 at.%. XRD and Raman analyses reveal a transition from a dense, columnar FCC NaCl-type solid solution with a (200) preferential orientation to a (111)-textured matrix containing secondary carbides (TiC, TaC) and sp^2^-bonded free carbon at elevated carbon levels. Nanohardness and elastic modulus reach an optimal plateau at ~35 at.% C (29 GPa and 350 GPa, respectively), followed by a decline to ~20 GPa and 223 GPa at 55 at.% C due to the percolation of soft carbon-rich phases. Remarkably, increasing carbon content drastically enhances tribological performance: the coefficient of friction decreases from 0.40 to 0.20, and the specific wear rate drops from 35 × 10^−6^ to 1.7 × 10^−6^ mm^3^/(N·m), consistent with a solid-lubrication mechanism inferred from as-deposited Raman trends and wear-track compositional analysis, though direct post-wear spectroscopic validation remains a priority for future work. Thermal stability assessments at 600 °C at an intermediate low pressure demonstrate excellent microstructural and mechanical retention for low-to-intermediate carbon compositions, with oxidation confined to a ~200 nm surface layer attributed to the formation of stable titanium and tantalum oxides and oxycarbides, which possibly forms an oxygen diffusion barrier at that temperature. An optimal carbon content of ~35 at.% C delivers a superior synergy of high hardness, exceptional wear resistance, and robust thermal stability, establishing (TiAlTaZrNb)Cx as a highly tunable coating system for next-generation protective applications. This work provides the first systematic composition–performance map for this quinary HEC system across a broad stoichiometric range, demonstrating that carbon stoichiometry serves as a master variable to tailor the balance between mechanical integrity and tribological functionality.

## 1. Introduction

High-entropy materials have fundamentally reshaped alloy design by leveraging configurational entropy to stabilize simple solid-solution phases amidst complex multi-principal-element chemistries. While bulk high-entropy alloys (HEAs) exhibit exceptional strength, damage tolerance, and environmental resistance [1], their widespread industrial adoption is often constrained by processing costs and bulk property limitations [2]. The compositional flexibility of HEAs, typically involving at least five principal elements with concentrations between 5 and 35 at.%, enables the formation of simple solid-solution phases and allows for effective tuning of their microstructures and properties [3,4].

In this context, high-entropy coatings have emerged as a highly efficient surface-engineering strategy, enabling the deposition of multi-component chemistries onto conventional substrates to impart extreme-environment functionality without compromising bulk integrity [5,6]. Since their introduction, high-entropy coatings have been fabricated using various techniques, including magnetron sputtering, electrodeposition, and laser-based methods, which also allow the incorporation of reactive elements such as nitrogen, oxygen, and carbon, leading to the formation of high-entropy nitrides, oxides, and carbides [7]. These coatings have demonstrated potential for applications requiring high wear resistance, corrosion protection, and thermal stability [8,9,10].

Among them, binary and ternary carbide-based coatings are particularly attractive due to their high hardness and low friction coefficients, which make them suitable for wear-resistant applications [11,12]. However, conventional binary and ternary carbides often face limitations under increasingly demanding conditions such as high temperatures, reduced lubrication, and severe mechanical loading. This has motivated the development of high-entropy carbide (HEC) coatings, which aim to combine high hardness with improved toughness and enhanced resistance to wear and oxidation.

The functional performance of HEC coatings is profoundly governed by carbon stoichiometry, which acts as a master variable dictating phase evolution, defect density, and bonding character. At sub-stoichiometric levels, carbon interstitials promote solid-solution strengthening and severe lattice distortion, typically maximizing hardness and elastic modulus [13,14,15]. However, as carbon content approaches and exceeds solubility limits, excess carbon precipitates as secondary carbides or segregates as sp^2^-bonded free carbon (amorphous/graphitic) at grain boundaries. While this phase separation can degrade mechanical integrity, it simultaneously introduces a potent solid-lubrication mechanism that drastically reduces friction and wear rates [16,17,18,19]. Consequently, identifying the optimal carbon window that balances intrinsic hardness with tribo-chemical lubrication remains a critical materials design challenge.

Recent investigations have begun to map this composition–property landscape in various HEC systems. Studies on refractory-rich carbides such as (Hf, Ta, Ti, V, Zr)C [16], (Cr, Hf, Mo, Ta, W)C [17], and (Ti, Zr, Nb, Ta, Fe)C [18] have demonstrated that carbon content critically modulates phase stability, with hardness typically peaking at intermediate concentrations before declining due to free-carbon percolation or secondary phase formation. Concurrently, elevated carbon levels consistently enhance tribological performance through the formation of carbonaceous tribofilms [19]. Despite these advances, the existing literature remains fragmented: most reports isolate mechanical or tribological responses, rarely integrating thermal stability or post-deposition microstructural evolution into a unified structure–property framework. Moreover, systematic evaluations across a broad carbon stoichiometry range in refractory-rich systems that combine strong carbide formers (Ti, Ta, Zr, Nb) with lattice-modifying and entropy-enhancing elements (like Al) are notably scarce.

To address these gaps, this study presents a comprehensive investigation of (TiAlTaZrNb)Cx high-entropy carbide coatings synthesized via reactive unbalanced-field pulsed-bias magnetron sputtering [20]. While the general trends of hardness optimization and carbon-mediated lubrication are established in HEC systems, the mechanistic novelty of this work lies in establishing the first systematic composition–performance map across a broad stoichiometric range (24–55 at.% C) for this quinary system. We explicitly link phase evolution, near-surface oxycarbide formation, and tribofilm-mediated wear mitigation to carbon stoichiometry, moving beyond confirmatory observations to provide a unified structure–property framework. The selected quinary metal composition was deliberately engineered to maximize configurational entropy (theoretical DS_conf = R·ln(5) ≈ 1.61R for equiatomic ratios; actual values from EDS-derived metallic fractions range between 1.54R and 1.59R, confirming operation in a high-entropy regime > 95% of the theoretical maximum) while combining refractory elements that thermodynamically stabilize the NaCl-type carbide lattice with aluminum, which enhances atomic size mismatch, promotes lattice strain, and may contribute to surface passivation under oxidative conditions. By precisely tuning the methane precursor flow, we systematically vary the carbon content from ~24 to ~55 at.% and rigorously correlate stoichiometry with microstructural evolution, nanomechanical response (evaluated via the Oliver–Pharr method [21]), tribological behavior, and thermal stability up to 600 °C under intermediate low pressure. Particular attention is paid to the influence of deposition parameters on lattice parameter variations, the identification of carbon phases via Raman spectroscopy, and the thermodynamic rationale for surface oxidation and decarburization via CO/CO_2_ evolution. The objective of this work is to systematically investigate the influence of carbon stoichiometry on the microstructural evolution, mechanical response, tribological behavior, and intrinsic thermal stability of (TiAlTaZrNb)Cx coatings, with the aim of identifying an optimal carbon content that synergistically balances high hardness, exceptional wear resistance, and robust thermal stability for demanding protective applications.

## 2. Materials and Methods

### 2.1. Coating Deposition

**Figure 1:**
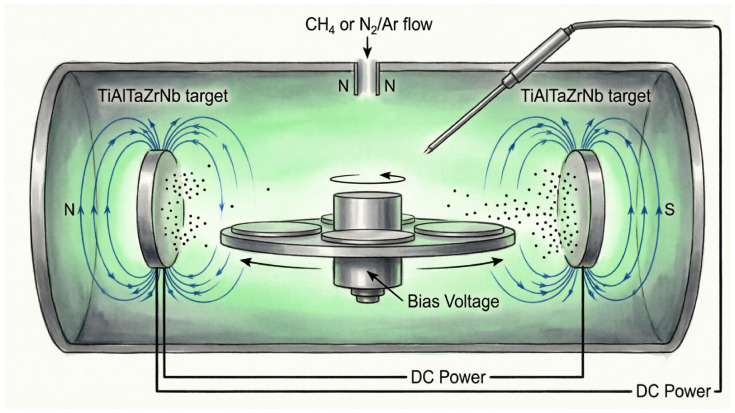
Schematic of the vacuum chamber used.

High-entropy (TiAlTaZrNb)Cx coatings were deposited onto AISI H13 hot-work steel and silicon (100) substrates by unbalanced-field pulsed-bias magnetron sputtering using an equiatomic preformed TiAlTaZrNb target of 99.9% purity from Able Target Limited (Nanjing, China). A schematic of the custom-made vacuum chamber used is shown in Figure 1. The steel samples (20 mm in diameter and 4 mm thick) were prepared by roughing with SiC sandpaper (grain size range 240–1500) and then polished to a mirror finish using a cloth in an aqueous suspension of powdered alumina with a grain size of 0.3 µm. Silicon (100) substrates were also used. Both silicon and steel substrates were degreased in an ultrasonic bath in an alcohol and acetone solution for 20 min. They were then positioned inside the vacuum chamber 90 mm from the rectangular target (500 mm height × 100 mm width × 5 mm thickness).

The chamber was initially evacuated to a base pressure of 0.04 Pa, and the substrates were then cleaned with an argon ion plasma at a pressure of 4 Pa and −650 V for 30 min. The deposition parameters are shown in Table 1. The bias voltage, power supplied to the target, process pressure, and temperature were kept constant, while the methane flow rate was varied. The magnetron source was operated in pulsed DC (pulsed direct current) mode. The unbalanced magnetic field configuration was used to synthesize the coatings. The argon flow rate was fixed at 65 sccm, and methane flow rates of 6, 9, and 12 sccm were used, corresponding to CH_4_/(Ar+CH_4_) ratios of 8.45%, 12.16%, and 15.58%, respectively. All gas flows were controlled by mass flow controllers (MFCs, model GFC de Aalborg) with an accuracy of ±1.5% of the full scale.

The deposition was carried out in reactive mode. No hysteresis control was implemented. As shown in Section 3.1, a gradual decrease in deposition rate with increasing CH_4_ flow was observed, which is consistent with the onset of target surface compound formation (i.e., poisoning). It is important to note that in reactive sputtering of carbides, the relationship between target surface composition and film stoichiometry is complex. While complete target poisoning would be expected for deposition of stoichiometric compounds from compound targets, in our reactive process with metallic targets, the deposition occurs in a compound-forming mode where both the target surface and the growing film undergo reaction with the reactive gas. The gradual, rather than abrupt, decrease in deposition rate suggests that the target operates in a transition regime where compound formation on the target surface is significant but not complete. Therefore, some degree of target poisoning cannot be ruled out, particularly for the highest CH_4_ flow (12 sccm), which may contribute to the reduced deposition rate and the preferential incorporation of aluminum [22]. However, the exact target surface condition (degree of poisoning) cannot be determined from our ex situ measurements. Future in situ diagnostics (e.g., optical emission spectroscopy) would be required to precisely quantify the target surface state.

A resistive heating system and a type K thermocouple, both mounted inside the vacuum chamber, were used to heat the substrate and measure its temperature, respectively, which was kept at a constant value of 180 °C. A pulsed bias voltage of −90 V (0.3 A) was applied to the substrates. The pulse parameters consisted of an active time (ON) of 80 µs and an inactive time (OFF) of 5 µs, corresponding to a pulse frequency of approximately 11.8 kHz and a duty cycle of 94.1% (calculated as ON/(ON + OFF)). These pulse conditions were selected to minimize the bias inactive time and thereby achieve a greater positive effect of the bias voltage on the microstructure and properties of the coatings, based on previous experience with similar coating systems [20]. The target power was set at 1700 W (470 V and 3.6 A). During deposition, the substrates were rotated at a constant speed of 12 rpm to ensure uniform coating thickness and composition across the sample surface.

Before depositing the (TiAlTaZrNb)Cx carbide, an adhesion layer of the high-entropy metallic alloy (same TiAlTaZrNb composition without carbon) was deposited for 10 min using the same conditions described in Table 1 for the bulk coating, resulting in a thickness of ~170 nm. While this interlayer is intended to promote coating–substrate bonding, quantitative adhesion testing (e.g., scratch or pull-off tests) was not performed in this study and will be addressed in future work.

### 2.2. Coating Characterization

#### 2.2.1. Microstructure, Composition and Phase Analysis

The surface and cross-sectional areas of the coatings were evaluated using a FIB-FESEM Scios 2 LoVac field emission scanning electron microscope (Thermo Fisher Scientific, Hillsboro, OR, USA) with a 5.0 kV high voltage and a secondary electron detector. Their elemental chemical composition was determined by energy-dispersive X-ray spectroscopy (EDS) using the integrated UltraDry 129 eV EDS unit (Thermo Fisher Scientific, Hillsboro, OR, USA). EDS area measurements were performed in triplicate at 10 kV, and gold coating of samples was not required. Given the known challenges of accurate carbon quantification by EDS in carbide coatings, the carbon contents reported in Table 2 should be considered semi-quantitative; however, it is a technique accepted by the scientific community.

Microstructural and phase analysis of the coating was performed on a PANalytical X’Pert PRO X-ray diffractometer (XRD) (PANalytical, Almelo, The Netherlands) using a Pixel 3D detector, a Cu Kα source with λ = 1.540598 Å, 45 kV, 40 mA, a 0.02° step and a time per step of 50 s. Diffractograms were recorded in the 2θ range of 20° to 80° using conventional θ–2θ geometry. Crystallite size was calculated using the Debye–Scherrer equation with HighScore Plus software (version 3.0c) and using the form factor 0.9.

For average grain size determination, surface images obtained by scanning electron microscopy (FESEM) as described above were used and manually analyzed with ImageJ (version 1.53e) and processed according to a normal distribution in Origin.

Micro-Raman spectroscopy analysis (Labram HR Evolution Confocal Raman Microscope, of HORIBA Scientific, Loos, France) was performed at room temperature using a 532 nm laser with a power of 50 mW, an acquisition time of 7 s, and an accumulation time of 5 s. Spectra were recorded in the range of 200 to 2000 cm^−1^. The analysis was performed for the identification of amorphous/graphitic carbon phases (D and G bands) and the vibrational modes of metal carbides present in the solid solution of the high-entropy carbide matrix.

The thickness and deposition rate were calculated in triplicate using the calotest technique with a 25 mm diameter steel sphere, rotating at 120 rpm for 1.45 min and using diamond paste as the abrasive. The thickness was calculated using Equation (1):

(1) $$t=\frac{x*y}{D}$$

where t is the coating thickness, y is the internal diameter of the indentation, x is the difference between the external and internal diameters of the indentation, and D is the diameter of the steel sphere used.

#### 2.2.2. Mechanical and Tribological Properties

The nanohardness and Young’s modulus of the coated samples were measured using a UNAT nanoindenter (ASMEC GmbH, Radeberg, Germany) with a Berkovich indenter by means of the Quasi-Continuous Stiffness Method (QCSM), applying a load of 10 mN, a dwell time of 19 s, and a frequency of 8.5 Hz to keep the maximum penetration depth within 10% of the coating thickness (1.9–2.5 µm), as can be seen later in the load–unload curves. A discharge load rate of 10% was applied. Fused silica was used as a reference sample to calibrate the nanoindenter. Ten indentations were made on each coated system, and the Oliver and Pharr method was used to deconvolute the hardness and elastic modulus [21].

The tribological behavior of the (TiAlTaZrNb)Cx coatings was evaluated in duplicate according to ASTM G99-17 using an Anton Paar CSM instruments TBR3 tribometer (manufactured by Aton Paar, Corcelles, Switzerland) in ball-on-disc mode with a 6 mm diameter alumina ball (Al_2_O_3_) as a counterbody. A normal load of 5 N was applied for 5.1 min for a sliding distance of 47.10 m. The radius of the wear track was 2.50 mm and the linear rotation speed was 25 mm/s at 585 rpm. The tests were carried out at an ambient temperature of 25 °C and a relative humidity of 74%. Tests were performed in duplicate; extrapolation to industrial environments with variable load, humidity, or counterbody material should be made with caution.

The profile of the wear track was determined using a Bruker DektakXT contact profilometer (Bruker Nano Surfaces and Metrology, Tucson, AZ, USA) and its cross-sectional area (*A*) was measured. The wear volume was calculated from Equation (2):

(2) V=A∗2π∗r

where *V* is the volume in mm^3^, *A* is the cross-sectional area of the wear track in mm^2^, and *r* is the radius of the wear track in mm. For this purpose, 12 wear profiles were measured along the circular wear track. The wear rate was estimated using Equation (3):

(3) $$k=\frac{V}{F*l}$$

where *k* is the wear rate in mm^3^/(N·m), *V* is the worn volume in mm^3^, *F* is the applied normal load in N, and *l* is the sliding distance in m.

The wear patterns were analyzed using surface imaging and EDS line spectroscopy with a Thermo Fisher Scientific Phenom XL G2 SEM microscope (of Thermo Fisher Scientific, Eindhoven, The Netherlands) operating at 15 kV with a secondary electron detector. Optical images of the counterbody (alumina ball) were obtained using a Keyence VK-X1000 3D laser profilometer and MultiFileAnalyzer VK-H1XME software version v2.1.217 (of Keyence Corporation, Osaka, Japan).

### 2.3. Thermal Stability of Coatings

A Fusion Factory low-pressure furnace (XERION Laboratories GmbH, Berlin, Germany) was used to anneal the silicon-coated samples at 600 °C at an intermediate low pressure to evaluate their intrinsic microstructural and mechanical stability with reduced oxygen presence. This temperature was selected as an initial assessment of thermal resistance, with the understanding that high-entropy carbides are typically evaluated at higher temperatures (800–1000 °C) under oxidizing conditions. The present study focuses on the intrinsic thermal stability of the coatings without the confounding effect of oxidation at low-pressure conditions; future work will address oxidation resistance under ambient atmosphere. The heat treatments were carried out at an intermediate low pressure of 5 × 10^−2^ mbar for 60 min using a heating rate of 5 °C/min and a cooling rate of 7 °C/min under nitrogen atmosphere (N_2_, 99.9% purity). After annealing, the cross-sectional microstructure and hardness of the coated silicon samples were analyzed by FESEM and nanoindentation, respectively.

## 3. Results

### 3.1. Microstructure and Phase Characterization

The elemental chemical composition of the synthesized (TiAlTaZrNb)Cx coatings, as determined by EDS/FESEM, is listed in Table 2. The carbon content increased from 23.7 at.% to 55.3 at.% with increasing methane flow rate from 6 sccm to 12 sccm. Oxygen levels below 3.3 at.% were detected, which are attributed to residual oxygen in the vacuum chamber and possible surface contamination during handling. Notably, the aluminum content increases with methane flow, while the concentration of refractory metals (Ti, Ta, Zr, Nb) decreases slightly. This behavior may be related to preferential target poisoning during reactive sputtering. Ti, Ta, Nb and Zr form stable carbide phases with lower sputtering yields than their metallic counterparts, whereas Al-containing surface compounds are generally less stable. Consequently, increasing methane flow may promote relative Al enrichment in the deposited coatings. Additionally, differences in sputtering yields, angular distribution of sputtered species, and scattering in the gas phase contribute to compositional differences between the target and the film. The variation in the relative concentrations of individual refractory metals (e.g., the decrease in Nb from 20.8 at.% in C6 to 9.6 at.% in C12) can be attributed to differences in carbide formation thermodynamics and sputtering rates of the metallic elements in the presence of increasing reactive gas. In summary, it should be clarified that the difference in the atomic content of the deposited coatings arises from target poisoning and the varying sputtering rates of the constituent metallic elements. The gradual target poisoning, particularly at high CH_4_ flow rates, reduces the deposition rate (Table 3) and modifies the film composition, favoring carbon incorporation and the formation of carbon-rich secondary phases (TiC, TaC, and graphite), as observed in the XRD and Raman patterns shown below.

It should be noted that carbon quantification by EDS in carbide coatings is subject to well-known uncertainties. The carbon contents reported in Table 2 should therefore be considered semi-quantitative, although the trend with increasing methane flow is reliable.

Table 3 shows the thickness and deposition rate of the deposited (TiAlTaZrNb)Cx coatings. Both parameters decrease with increasing methane flow for the same deposition time. This behavior is attributed to the reduction in the relative argon concentration in the gas mixture and probably to target poisoning by carbon, which decreases the sputtering rate and consequently the coating deposition rate [15].

**Figure 2:**
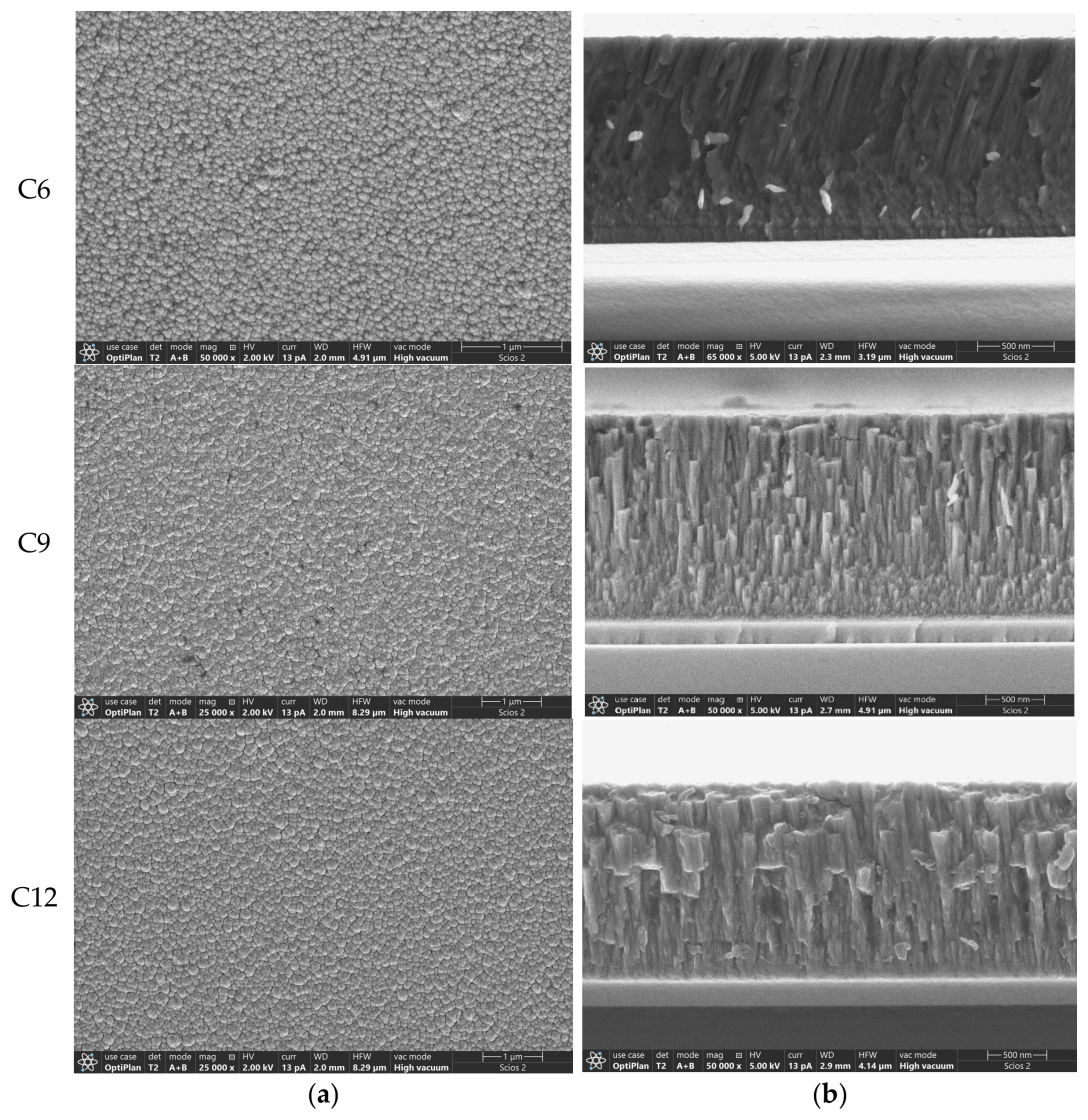
SEM coating images of C6, C9 and C12 deposited coatings: (**a**) top view, (**b**) cross-section.

Figure 2a shows surface images of the coatings deposited with different carbon contents. All coatings exhibit a cauliflower-like, dome-shaped morphology. Figure 2b shows cross-sectional images revealing a dense, homogeneous columnar growth structure. The column widths correspond reasonably with the grain sizes observed in the surface images. The C6 coating exhibits oblique columnar growth, the origin of which is not fully understood and requires further investigation.

**Figure 3:**
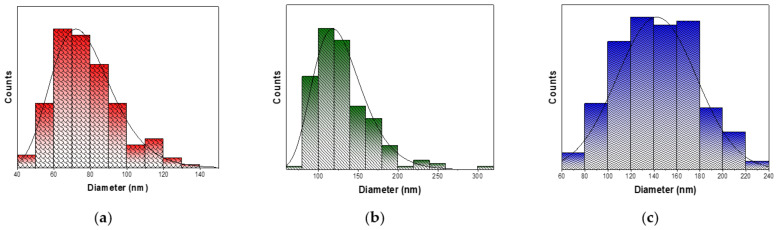
Average grain size of deposited coatings: (**a**) C6, (**b**) C9 and (**c**) C12.

The average grain sizes of the deposited coatings, determined from FESEM surface images using ImageJ software, were approximately 70 ± 10.5 nm for C6 (lowest carbon content), 130 ± 20.9 nm for C9, and 140 ± 25.3 nm for C12, as shown in Figure 3.

It should be noted that the XRD-derived crystallite size (coherently diffracting domains) decreases with increasing carbon content, while the SEM-measured columnar grain size increases. This apparent discrepancy is physically consistent: higher carbon incorporation introduces lattice strain and secondary phase nucleation, refining crystallite size, while enhanced surface diffusion and columnar coalescence during deposition promote wider morphological grains. These two metrics represent distinct structural length scales and do not contradict one another.

**Figure 4:**
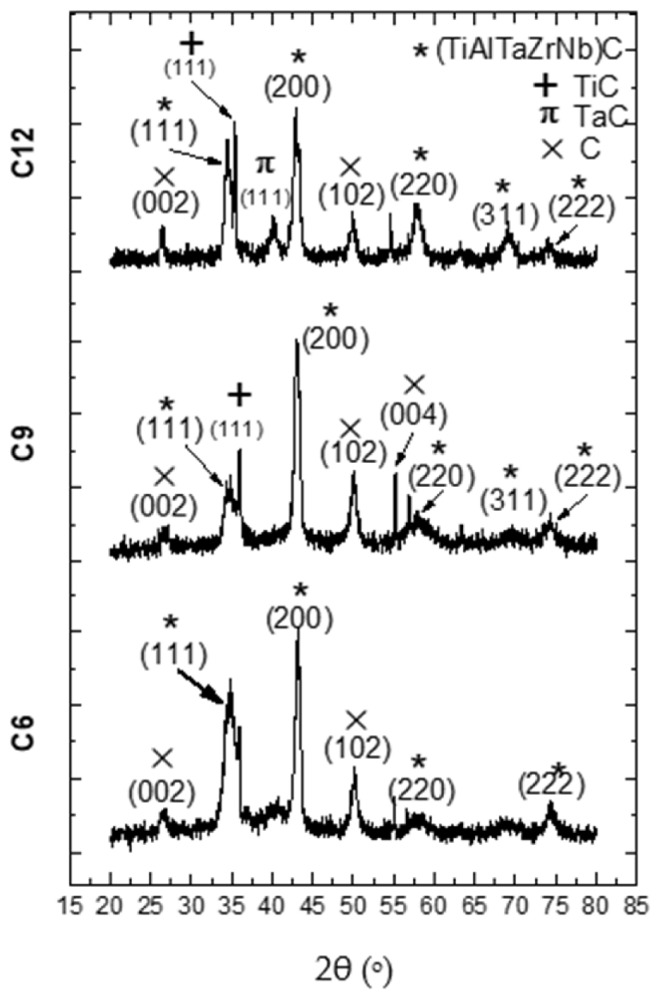
XRD diffraction patterns of deposited (TiAlTaZrNb)Cx coatings.

The XRD diffraction patterns of the deposited (TiAlTaZrNb)Cx coatings are shown in Figure 4. All patterns exhibit a face-centered cubic (FCC) NaCl-type structure characteristic of high-entropy carbides, with preferential growth in the (200) plane and, to a lesser extent, in the (111) plane. Additional peaks corresponding to TiC (111) at 35.5° (JCPDS card No. 65-8804), TaC (111) at 40° (JCPDS card No. 03-065-0282), and graphite are also observed.

Similar results were reported by Rahmadtulloh et al. [18], who deposited TiZrNbTaFeCx coatings with different carbon contents and observed that the preferred orientation changes from (200) to (111) as the carbon content increases, implying that the amount of carbon enhances the growth of the (111) plane and retards the growth of other orientations. Additionally, those researchers found that at low carbon contents, the coatings were amorphous, transitioning to a crystalline FCC structure as carbon content increased, accompanied by the formation of other carbides, including TiC. All coatings exhibit a peak associated with carbon (graphite); the carbon content is relatively low for samples C6 and C9 (sub-stoichiometric), contributing to the formation of TiC and subsequently to that of TaC in sample C12, which has the highest carbon content. The precipitation of binary carbides alongside the formation of the solid solution has been observed in high-entropy carbide coatings with low carbon contents [23,24]. Consequently, the C9 and C12 coatings are more accurately described as HEC-based multiphase nanocomposites rather than single-phase solid solutions.

Table 4 shows the crystallite size, interplanar distance values, and lattice parameters of the deposited high-entropy carbide coatings, calculated from the (200) peak of the XRD patterns using the Bragg and Debye–Scherrer equations. The (200) peak shifts from 43.0° for C6 to 43.2° for C9, and then to 42.9° for C12. These variations in lattice parameter (Table 4) may be influenced by changes in solid solution effects, phase composition, or residual stress state. However, since residual stresses were not directly measured, no causal link between peak shifts and stress magnitudes can be established.

Despite the small changes in lattice parameter, these changes can influence the mechanical and tribological properties of the coatings, as discussed later. Similar observations are found in the literature: Zhu et al. [22] reported that small changes in lattice parameter (from 0.42847 nm to 0.42785 nm) were accompanied by significant changes in residual stresses (from 0.41 GPa to 0.63 GPa) in TiCN coatings. Likewise, Rahmadtulloh et al. [18] observed that variations in lattice parameter (from 0.4457 nm to 0.4447 nm) correlated with compressive residual stresses ranging from −1.36 GPa to −0.99 GPa in TiZrNbTaFeCx coatings.

**Figure 5:**
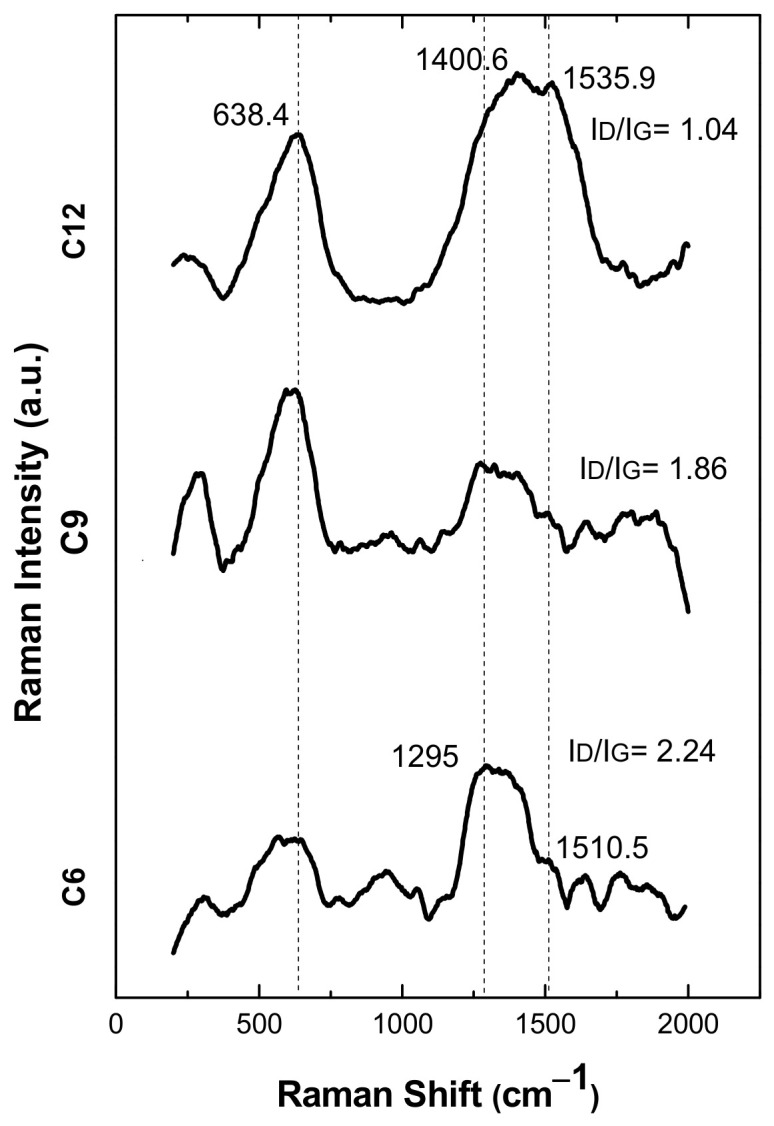
Raman spectrum patterns of the deposited (TiAlTaZrNb)Cx carbides.

Figure 5 shows the Raman spectra of the deposited (TiAlTaZrNb)Cx coatings. In all coatings, two intense peaks are observed: for sample C6, they are centered at 1295 cm^−1^ and 1510.5 cm^−1^, which shift to 1405.6 cm^−1^ and 1535.9 cm^−1^ for coating C12. These two peaks are attributed to the symmetric A_1g_ vibration mode of amorphous carbon (D band) and the symmetric E_2g_ vibration mode of graphitic carbon (G band), respectively.

The D and G bands increase in intensity with increasing carbon content, suggesting that higher methane flow results in a greater amount of carbon that does not react with the metals in the high-entropy alloy and remains as free carbon in the deposited coatings. The shift of the D and G peaks to higher wavenumbers, accompanied by a decreased I_D_/I_G_ ratio, indicates a higher content of free carbon with sp^2^ bonds and greater structural order. Additionally, peaks were observed in the 397–770 cm^−1^ range, corresponding to the A_1g_ and E_g_ vibrational modes of the metal carbides present in the solid solution of the high-entropy carbide matrix.

Despite the decrease in the I_D_/I_G_ ratio with increasing methane flow, particularly for C12, the hardness and Young’s modulus decrease, which is attributed to increased graphite formation and TiC/TaC precipitation, as discussed in Section 3.2. Nikitin et al. [25] found similar results when synthesizing carbides, nitrides, and carbonitrides of high-entropy Ti-Zr-Nb-Hf-Ta alloys by high-speed arc discharge plasma jet. They observed a peak centered at 1360 cm^−1^ assigned to graphite (D band), another at 1570 cm^−1^ from amorphous carbon (G band), and additional peaks between 500 and 700 cm^−1^ corresponding to metallic carbonitride bonds. Similar results were obtained by Kao et al. [26] in their Raman evaluations of CrNbSiTaZr high-entropy carbide coatings deposited by reactive RF magnetron sputtering. The Raman spectra exhibited two broad, intense peaks around 1390 cm^−1^ and 1563 cm^−1^, representing disordered (D-band) and graphitic (G-band) carbon structures. They also found that hardness decreases with increasing acetylene flow due to greater amorphous carbon or graphite phase formation in the coatings.

### 3.2. Evaluation of Mechanical and Tribological Properties

Table 5 shows the hardness and Young’s modulus of the deposited coatings as a function of carbon content. Both properties initially increased slightly with increasing carbon content, from 27.9 GPa to 28.7 GPa and from 275 GPa to 350 GPa for samples C6 and C9, respectively. However, given the overlap within experimental uncertainty, the hardness reaches a plateau/optimal window around ~35 at.% C rather than a sharp peak. For sample C12 (55.3 at.% C), both properties decreased to 20.0 GPa and 223 GPa, respectively.

The increase in hardness from C6 to C9 results principally from solid solution strengthening associated with a higher packing factor of the high-entropy carbide solid solution, higher configurational entropy of the coating, as well as greater lattice distortion of the crystal lattice. The formation of secondary phases in sample C12, such as TiC, TaC, graphite, and possibly amorphous carbon (as observed by XRD and Raman), led to a reduction in the mixing entropy and a decrease in hardness. In addition to target poisoning, the reduction in the Ar/CH_4_ ratio with increasing methane flow also decreases the intensity of argon ion bombardment during deposition, which could affect the lattice parameter and defect structure of the coatings. However, the dominant factors affecting the mechanical properties remain the phase composition and the presence of soft carbon-rich phases. While residual stress variations cannot be quantified from the XRD data alone, the observed lattice parameter changes (Table 4) may be related to differences in the coating microstructure and phase composition, which in turn influence the mechanical properties. However, the dominant factors affecting the hardness decrease in C12 remain the phase composition and the presence of soft carbon-rich phases.

On the other hand, the C12 coating exhibited the smallest thickness (1.9 µm, Table 3), which may influence its mechanical response, although thickness alone is not a direct indicator of residual stress state. These statements are consistent with the observations of other authors [26,27,28], who deposited high-entropy carbides of (CrNbSiTaZr), (TiCrZrVNb), and (CrNbSiTiZr), respectively. While it is true that variations in the crystal lattice correlate with changes in hardness, the exact and significant contribution of residual compressive stress remains unquantified due to measurement limitations encountered in this study. We acknowledge that the lack of direct residual stress measurements prevents us from definitively deconvoluting the contributions of solid solution strengthening, grain size effects, and residual stress to the observed hardness variations. Future studies employing techniques such as X-ray diffraction sin^2^ψ or substrate curvature measurements would be valuable to quantify the role of residual stresses.

Table 5 also presents the H^2^/E and H^3^/E^2^ ratios, which are indicators of elastic deformation capacity and resistance to plastic deformation, respectively. Sample C6 exhibited the highest values for both ratios (H^2^/E ≈ 2.81, H^3^/E^2^ ≈ 0.28), suggesting theoretically better tribological behavior based solely on elastic-plastic parameters. However, it is well established that wear resistance is influenced by multiple factors beyond these ratios, including microstructure, phase composition, coefficient of friction, and the presence of lubricious phases such as graphite or amorphous carbon. As will be discussed below, the actual wear performance is governed by a complex interplay of these factors. Nonetheless, all coated systems exhibited superior mechanical properties compared to the uncoated AISI H13 steel substrate, which showed a hardness of 5.5 GPa and a Young’s modulus of 210 GPa.

**Figure 6:**
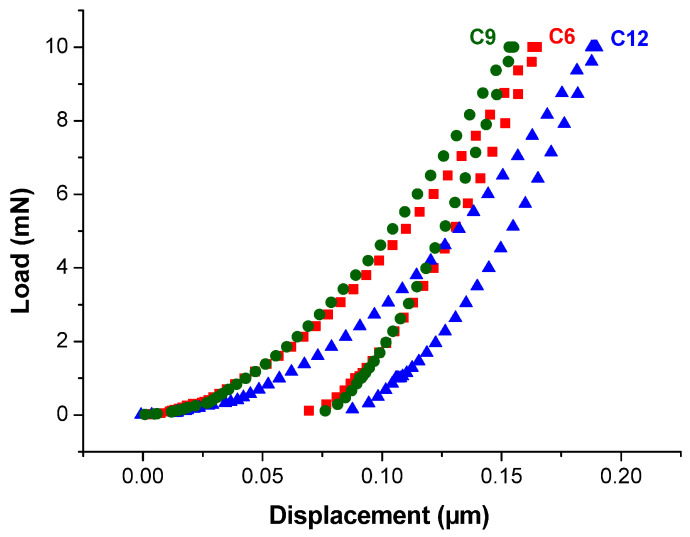
The load–displacement curves of the deposited coatings.

Figure 6 shows the load–displacement curves of the three coatings. The C9 coating exhibited the lowest penetration depth under the same maximum load (10 mN), which correlates with its highest hardness (28.7 GPa) and greater resistance to plastic deformation. In contrast, the C12 coating showed the highest penetration depth, consistent with its lower hardness (20.0 GPa) and higher susceptibility to permanent deformation.

The slope of the unloading segment of each curve is related to the elastic modulus of the coating. The steeper unloading slopes observed for C6 and C9, compared to C12, indicate higher stiffness and greater elastic recovery. This behavior is consistent with the Young’s modulus values reported in Table 5 (275 GPa and 350 GPa for C6 and C9, respectively, versus 223 GPa for C12).

Although C9 presented the highest hardness and C6 exhibited the best H/E and H^3^/E^2^ ratios (suggesting superior theoretical wear resistance), the actual tribological performance is influenced by additional factors, as discussed in the following section.

**Figure 7:**
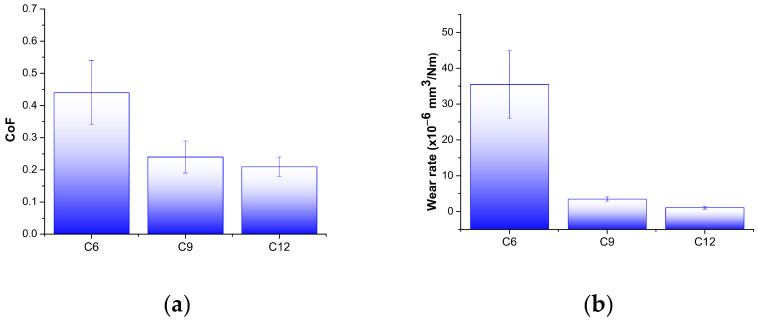
Coefficient of friction (**a**) and wear rate (**b**) of (TiAlTaZrNb)Cx coatings.

Figure 7 illustrates a systematic reduction in both the coefficient of friction (CoF) and the specific wear rate with increasing carbon content. The CoF decreases from ~0.40 for the C6 coating to ~0.20 for C12, while the wear rate drops by nearly one order of magnitude, from 35 × 10^−6^ to 1.7 × 10^−6^ mm^3^/(N·m). This pronounced tribological enhancement is primarily driven by the solid-lubricating action of carbon-rich phases (graphitic and amorphous carbon) that segregate to grain boundaries and the sliding interface. This mechanism is directly corroborated by Raman spectroscopy (Figure 5), where the progressive decrease in the I_D_/I_G_ ratio with higher methane flow indicates an evolution toward more ordered sp^2^-bonded graphitic domains. The concurrent reduction in I_D_/I_G_ ratio and wear rate strongly supports the hypothesis that increasingly ordered carbon structures facilitate the in situ formation of a low-shear-strength tribofilm during sliding, which effectively mitigates abrasive and adhesive wear. Although the precipitation of secondary carbides (TiC, TaC) at elevated carbon levels marginally reduces the configurational entropy of the high-entropy solid solution, the dominant role of the lubricious carbon network ultimately governs the superior wear resistance observed for the C12 coating.

**Figure 8:**
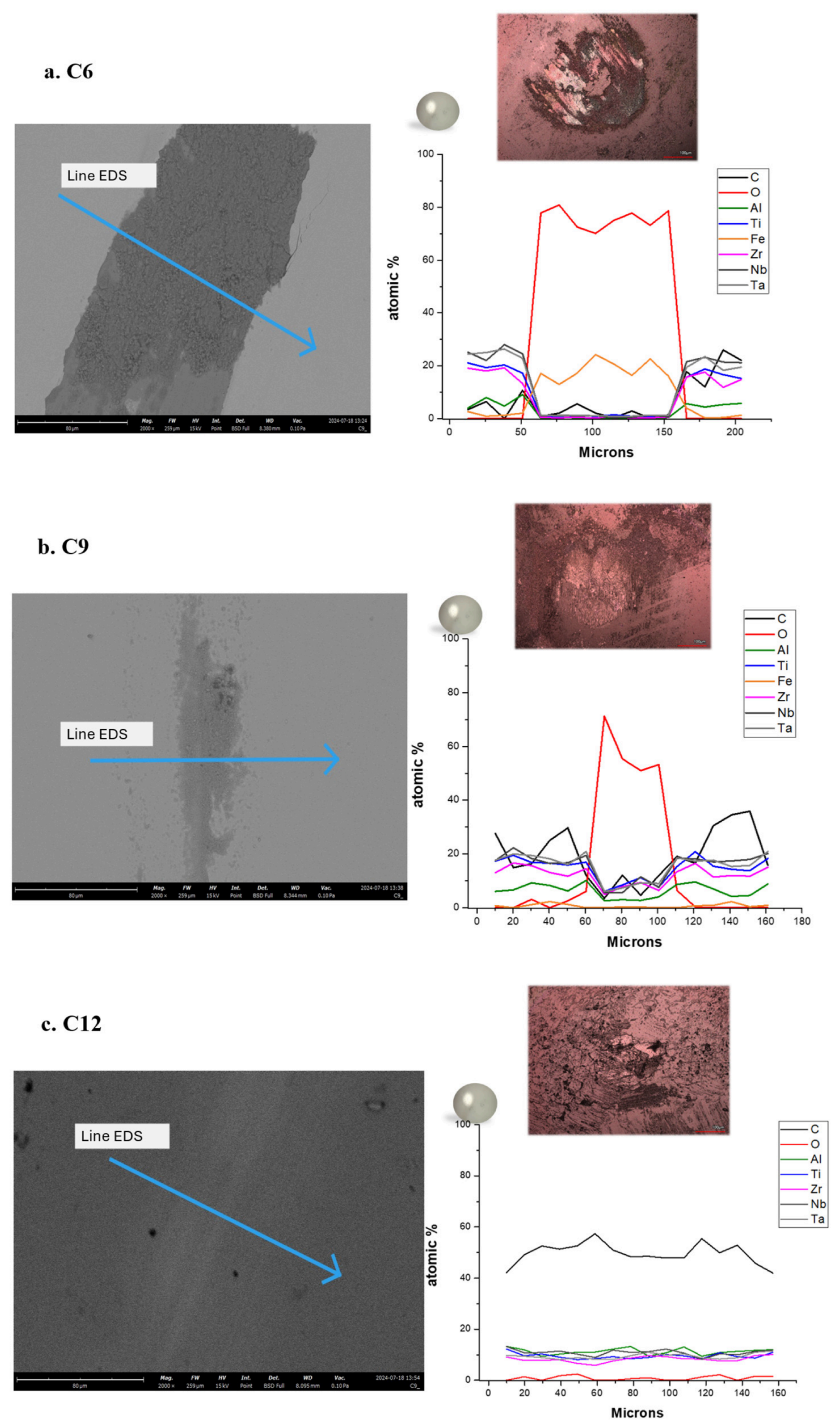
Surface SEM images of the wear tracks of the deposited (TiAlTaZrNb)C_X_ coatings and their corresponding line EDS spectra, together with optical images of the alumina counterbody for samples: (**a**) C6, (**b**) C9, and (**c**) C12.

Figure 8 shows SEM images of the wear tracks, elemental chemical composition determined by line EDS, and optical images of the alumina counterbodies for the three (TiAlTaZrNb)Cx coatings.

For the C6 coating, the SEM image (Figure 8) shows delamination and microcracks along the wear track path, characteristic of mixed wear modes. The line EDS profile reveals increased oxygen and iron content in the wear zone, indicating high oxidation (tribo-oxidation) and exposure of the steel substrate (iron signal from AISI H13). The optical image of the counterbody shows significant wear and adhered coating material. Overall, the C6 coating exhibits a combination of tribo-oxidative, adhesive, and abrasive wear modes.

For the C9 coating, the SEM surface image shows less pronounced wear than C6, with abrasive and adhesive wear but no delamination or peeling of the coating from the steel substrate. Some wear and adhered coating material are observed on the alumina ball. However, the high oxygen content in the wear pattern of this coating also suggests some degree of tribo-oxidative wear.

The C12 coating exhibits a very imperceptible wear pattern in the SEM image. In the line EDS profile, neither oxygen nor iron is visible, indicating that the steel substrate was not exposed during the tribological test. These observations are consistent with its low coefficient of friction and wear rate shown in Figure 7.

While direct spectroscopic analysis of the wear track (e.g., Raman or XPS) would provide definitive confirmation of tribofilm chemistry, the combined evidence from EDS line profiling (complete suppression of Fe/O signals), the strong correlation between the decreasing I_D_/I_G_ ratio and CoF reduction, and established literature on carbon-rich HEC systems strongly supports the formation of a low-shear-strength carbonaceous tribofilm. Future work will prioritize in situ or post-wear spectroscopic mapping to directly validate this mechanism.

The delamination observed in the C6 coating highlights the importance of interfacial toughness. While a metallic interlayer was employed to enhance adhesion, quantitative scratch/adhesion testing was beyond the scope of this study. Future work will include standardized adhesion evaluation to correlate interfacial strength with carbon stoichiometry and wear performance.

Table 6 summarizes the mechanical and tribological properties of high-entropy carbide coatings reported in selected studies from the literature, together with the results obtained in the present work.

The evolution of mechanical and tribological properties with increasing carbon content follows a consistent trend across all studies: hardness initially increases up to an optimal carbon concentration, and then decreases due to excess free carbon (amorphous or graphitic) precipitating at grain boundaries. Concurrently, the coefficient of friction and wear rate decrease with higher carbon content, demonstrating the solid lubrication effect of carbon-rich phases. Kao et al. [24,27] also observed that coatings with the highest carbon content exhibited the lowest wear rates, despite reduced hardness.

Quantitative differences exist among studies. Jhong et al. [26] reported significantly lower wear rates (~10^−7^ mm^3^/(N·m)) compared to our work (~10^−6^ mm^3^/(N·m)), while Kao et al. [27] achieved higher initial hardness (~37 GPa) than our maximum (~29 GPa). These variations are likely due to differences in coating composition, deposition technique, testing conditions, or counterbody material. Nevertheless, the overall trend confirms that carbon content is a key parameter for tuning the balance between hardness and tribological performance in high-entropy carbide coatings.

### 3.3. Thermal Stability Study

The (TiAlTaZrNb)Cx coatings deposited onto silicon (100) substrates were annealed at 600 °C for 60 min at a pressure of 5 × 10^−2^ mbar and subsequent cooling under a nitrogen atmosphere (99.9% purity) to evaluate their thermal stability. Table 7 presents the hardness and Young’s modulus values measured one day after annealing.

Considering the standard deviations, coatings C6 and C9 maintained their hardness and elastic modulus after annealing (27.9 ± 3.17 GPa vs. 27.9 ± 1.1 GPa as-deposited for C6; 26.6 ± 3.11 GPa vs. 28.7 ± 1.6 GPa as-deposited for C9). In contrast, coating C12 showed a slight decrease in hardness from 20.0 ± 1.0 GPa (as-deposited) to 15.4 ± 1.17 GPa after annealing, accompanied by a reduction in Young’s modulus from 223.4 ± 15.2 GPa to 184.2 ± 14.5 GPa. The H^2^/E and H^3^/E^2^ ratios of C12 decreased after annealing (from 1.79 to 1.29, and from 0.16 to 0.11, respectively), indicating a reduction in its elastic deformation capacity and resistance to plastic deformation, consistent with the observed decrease in hardness. Despite this reduction, the coating remained structurally stable and no delamination was observed.

**Figure 9:**
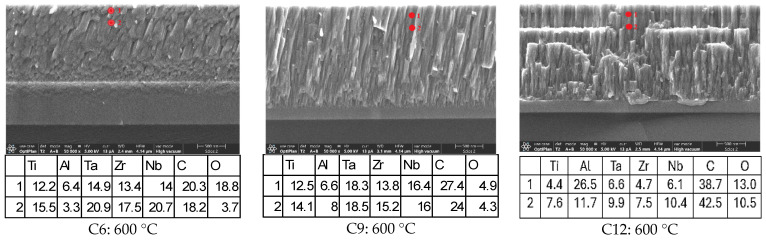
Cross-sectional SEM images of the coatings C6, C9 and C12 annealed at 600 °C.

Figure 9 shows cross-sectional SEM images of the three coatings after annealing at 600 °C. All coatings retained their columnar structure, and no visible oxide layer was observed on their surfaces. The stepped fracture pattern observed in the cross-section of the C12 coating is particularly notable and may be related to the presence of secondary phases (TiC, TaC, graphite) at grain boundaries, which can act as stress concentrators during brittle fracture of the silicon substrate.

EDS analysis performed at two points (Point 1 at ~100 nm from the surface, Point 2 at ~300 nm into the bulk) revealed that the oxygen content near the surface was higher than in the bulk for all coatings, indicating that oxidation was limited to the near-surface region (first ~200 nm). This superficial oxidation did not significantly deteriorate the microstructure or the mechanical properties of the coatings, as confirmed by the SEM images and hardness values.

The increased oxygen and carbon content (no carbon depletion observed) near the surface after annealing at 600 °C at an intermediate low pressure can be explained as follows: At temperatures below 500 °C, oxygen atoms diffuse into the near-surface region of the HEC lattice, occupying interstitial sites and partially substituting for carbon atoms. This leads to the formation of a protective oxycarbide layer (e.g., (Ti,Ta,Zr,Nb)(C,O)) that limits further inward oxygen diffusion. Notably, the formation of this oxycarbide phase incorporates carbon into the surface lattice, which accounts for the observed carbon enrichment rather than depletion in the near-surface region. At higher temperatures (not reached in this study), the formation of stable metal oxides such as TiO_2_, Ta_2_O_5_, Nb_2_O_5_, and Al_2_O_3_ would occur, and a further temperature increase could lead to the evolution of CO and CO_2_, thereby promoting coating oxidation [30,31]. According to thermochemical data for transition metal carbides [32], the equilibrium partial pressure of CO over MC phases at 873 K is extremely low under conditions of low residual oxygen impurities (<1 ppm O_2_ in N_2_) and surface-adsorbed H_2_O. Therefore, significant decarburization via gaseous species does not occur at the tested temperature, and the near-surface carbon enrichment is consistent with the formation of a stable oxycarbide phase rather than carbon loss.

In summary, the (TiAlTaZrNb)Cx coatings with low to intermediate carbon content (C6 and C9, ~24–35 at.% C) exhibited good intrinsic thermal stability at 600 °C, maintaining their columnar microstructure and hardness. The high-carbon coating (C12, ~55 at.% C) showed a moderate reduction in hardness (from 20.0 GPa to 15.4 GPa) but remained structurally stable. It should be noted that this assessment reflects microstructural and mechanical stability at intermediate low pressure and reduced oxygen presence. The oxidation resistance of these coatings at higher temperatures (800–1000 °C) under ambient atmosphere requires further investigation. Nonetheless, the optimal carbon content for balanced as-deposited and post-annealing performance is approximately 35 at.%.

Under fully atmospheric, highly oxidizing conditions, the degradation mechanisms would likely be more severe, including (1) enhanced formation of volatile oxide species (particularly WO_3_ and MoO_3_ if present, though these are not in our composition); (2) accelerated internal oxidation and formation of complex oxide scales; (3) potential carbon loss through CO/CO_2_ evolution at higher temperatures; and (4) possible spallation due to thermal expansion mismatch between the oxide scale and the underlying carbide coating. However, the presence of Al in our coating may promote the formation of a protective Al_2_O_3_ layer under oxidizing conditions, potentially improving oxidation resistance compared to Al-free refractory carbide systems. Future work will address oxidation resistance under ambient atmosphere to provide a more complete assessment for practical deployment.

## 4. Conclusions

This study successfully achieved its objective of systematically correlating carbon stoichiometry with the microstructural evolution, mechanical response, tribological behavior, and intrinsic thermal stability of (TiAlTaZrNb)Cx high-entropy carbide coatings. The key findings and novel contributions of this work are summarized as follows:

An optimal carbon window of ~35 at.% was identified, delivering a superior balance of high nanohardness (~29 GPa), significantly reduced wear rate (decreasing from ~35 × 10^−6^ to ~1.7 × 10^−6^ mm^3^/(N·m) across the series), and robust thermal stability up to 600 °C.

Increasing carbon content dramatically improves tribological performance through the in situ formation of solid-lubricating carbonaceous tribofilms. While direct post-wear spectroscopic analysis (Raman/XPS) was beyond the scope of this study, the strong correlation between the decreasing I_D_/I_G_ ratio, reduced CoF, and suppressed substrate exposure strongly supports this mechanism. Future work will prioritize direct wear-track spectroscopy to definitively validate tribofilm chemistry.

Coatings with low-to-intermediate carbon content (~24–35 at.% C) maintain their columnar microstructure and mechanical integrity after annealing at 600 °C under low pressure, with oxidation strictly confined to a ~200 nm near-surface layer. The behavior under fully atmospheric, highly oxidizing conditions remains to be evaluated and will be addressed in future studies to assess practical deployment viability.

The structural evolution transitions from a dominant HEC matrix at lower carbon levels to a multiphase nanocomposite containing secondary carbides (TiC, TaC) and sp^2^-bonded free carbon at higher concentrations. This phase distribution, rather than a single-phase solid solution, critically governs the mechanical softening and tribological enhancement at elevated carbon contents.

Finally, while lattice parameter variations correlate with hardness trends, the exact contribution of compressive residual stress remains unquantified due to measurement limitations. Future investigations employing XRD sin^2^ψ or substrate curvature techniques will be essential to fully deconvolute stress-mediated strengthening.

## Figures and Tables

**Table 1:** Deposition parameters of the (TiAlTaZrNb)Cx coatings.

Bias (V)	Temp. (°C)	Pressure (Pa)	Ar-Flow (sccm)	Power (W)	Bias Pulse ON/OFF Time (μs)
−90	180	0.45	65	1700	80/5
Sample		C6	C9		C12
CH_4_ flow (sccm)		6	9		12
Deposition time (min)		180	195		210

**Table 2:** Elemental composition of (TiAlTaZrNb)Cx coatings [at.%].

Sample	Ti	Al	Ta	Zr	Nb	C	O
C6	14.6 ± 0.4	5.6 ± 0.4	15.7 ± 0.5	16.3 ± 0.5	20.8 ± 0.5	23.7 ± 1.0	3.3 ± 0.1
C9	13.2 ± 0.7	6.5 ± 0.4	12.4 ± 0.9	13.1 ± 0.8	16.4 ± 1.0	35.0 ± 1.5	3.3 ± 1.4
C12	8.0 ± 0.1	9.2 ± 0.1	7.0 ± 0.1	7.8 ± 0.1	9.6 ± 0.2	55.3 ± 0.5	2.9 ± 0.1

**Table 3:** Thickness and deposition rate of (TiAlTaZrNb)Cx coatings.

Sample	Thickness (µm)	Deposition Rate (nm/min)
C6	2.3 ± 0.1	12.8
C9	2.5 ± 0.1	12.7
C12	1.9 ± 0.1	9.0

**Table 4:** Crystallite size, interplanar distance values, and lattice parameters of the deposited high-entropy carbide coatings.

Coating	FWHM	Crystallite Size (nm)	d (200) (nm)	Lattice Parameter (nm)
C6	0.73	12.23	0.2102	0.4203
C9	0.77	11.59	0.2092	0.4185
C12	0.90	9.92	0.2106	0.4213

**Table 5:** Hardness, Young’s modulus and H/E ratios of (TiAlTaZrNb)Cx coatings.

Coating	H	E (GPa)	H^2^/E	H^3^/E^2^
C6	27.9 ± 1.1	275.0 ± 40.0	2.83	0.29
C9	28.7 ± 1.6	350.1 ± 39.5	2.35	0.19
C12	20.0 ± 1.0	223.4 ± 15.2	1.79	0.16

**Table 6:** Comparison of mechanical and tribological properties of HEC coatings from different studies.

Reference	Coating System	Deposition Method	C Content	Hardness (GPa)	CoF	Wear Rate (mm^3^/(N·m))
Kao et al. [26]	CrNbSiTaZrCx	RF magnetron sputtering	Increasing with C_2_H_2_	37.4 → 14.4	Decreases with C	Lowest at highest C
Xu et al. [27]	(TiCrZrVNb)C	Multi-arc ion plating	Variable (R_C)	10.7 → 27.1 → 11	n.d.	Decreases with C
Jhong et al. [28]	(CrNbSiTiZr)Cx	Magnetron sputtering	36.7 → 87.8 at.%	32 → 23	0.4 → 0.07	3.2 × 10^−7^ → 2 × 10^−7^
Kao et al. [29]	TaNbSiZrCrCx	RF magnetron sputtering	0 → 77.6 at.%	Decreases with C	Decreases with C	Lowest at highest C
This work	(TiAlTaZrNb)Cx	Magnetron sputtering	24 → 55 at.%	28 → 29 → 20	0.4 → 0.2	35 × 10^−6^ → 1.7 × 10^−6^

**Table 7:** Hardness and modulus of elasticity of the annealed (TiAlTaZrNb)Cx coatings deposited onto silicon (100) substrates.

Coating	H (GPa)	E (GPa)	H^2^/E	H^3^/E^2^
C6 600 °C	27.9 ± 3.17	247.1 ± 25.5	3.15	0.36
C9 600 °C	26.6 ± 3.11	316.1 ± 35.8	2.24	0.19
C12 600 °C	15.4 ± 1.17	184.2 ± 14.5	1.29	0.11

## Data Availability

The data presented in this study are available upon request from the corresponding author.

## Abbreviations

The following abbreviations are used in this manuscript:

AISI	American Iron and Steel Institute
ASTM	American Society for Testing and Materials
BCC	Body-centered cubic
CoF	Coefficient of friction
EDS	Energy-dispersive X-ray spectroscopy
FCC	Face-centered cubic
FESEM	Field emission scanning electron microscopy
FIB-FESEM	Focused ion beam–field emission scanning electron microscopy
FWHM	Full width at half maximum
HEA	High-entropy alloy
HEC	High-entropy carbide
JCPDS	Joint Committee on Powder Diffraction Standards
MFC	Mass flow controller
pDC	Pulsed direct current
QCSM	Quasi-continuous stiffness method
SEM	Scanning electron microscopy
SiC	Silicon carbide
XRD	X-ray diffraction
